# 

**Published:** 1916-07-01

**Authors:** 


### Passing Events and Topics.

### £330 from a Sale of Work.

The annual sale of articles made by the invalids in the Hospital for Incurables, Mauldeth, Manchester, held recently, realised over £330.

### East London Hospital for Children.

The Convalescent Home of the hospital at Bognor has not been handed over for the use of the wounded, as stated in the Special Hospital Sunday Number, but has been commandeered by the Government for the billeting of soldiers.

### British Pharmaceutical Conference.

The annual meeting of the British Pharmaceutical Conference will take place on July 11 in the Lecture Theatre of the Pharmaceutical Society, 17 Bloomsbury Square, London, W.C. Like last year, the meeting this year will be of a formal character, and no scientific papers will be read. The presidential address will be delivered by Dr. David Hooper.

### Glamorganshire's 27 Red Cross Hospitals.

A generous gift to the Red Cross Society has added another auxiliary hospital to the number now at work in Glamorganshire, making a total of twenty-seven. The new building, known as St. Winifred's, Dinas Powis, is the benefaction of Mr. T. P. Thomas and Mrs. Thomas, of Merevale. It has accommodation for forty patients and has been equipped in the most up-to-date manner. Mrs. Thomas will act as matron.

### HOSPITAL RESULTS IN 1915.

**City of London Hospital for Diseases of the Chest, Victoria Park.**—A neat report with an attractive cover which invites interior inspection. Totals of in-patients and out-patients were each smaller, but the average stay of in-patients was slightly longer. A number of soldiers have received treatment, including some discharged as unfit for further military service. The tuberculosis dispensary for the treatment of patients from the Metropolitan Borough of Hackney is in connection with the hospital, but it is anticipated that no cost will fall upon the institution in relation to this department. Total income of the hospital for 1915, £13,823 (including £1,145 legacies). Total expenditure, £14,505.

**Hounslow Hospital.**—As a memorial to two of the founders of the hospital, the Rev. H. Layton and Dr. H. Sydney—whose deaths were recorded in last year's report—a special fund has been raised to build a nursing home, the construction of which will shortly be commenced. The average weekly cost of each in-patient has fallen from £1 1s. 10d. to 19s. 11d. The numbers of in- and out-patients have both increased.

**Metropolitan Hospital, Kingsland Road.**—The accommodation for in-patients, including provision for soldiers, is increased to three times its former size; this has been effected by taking in an adjacent London County Council School for a military section and converting the nurses' old quarters into two wards. The nurses are now housed in six different buildings, an arrangement which must be regarded as of a temporary character. A tuberculosis dispensary and beds for four cases of consumption, for diagnosis and observation, have been provided. The total income (including £3,158 for military cases and £280 legacies) was £16,696, and the total expenditure £19,331. We notice that the names of chief members of the nursing staff are recorded in the report.

**St. John's Hospital for Diseases of the Skin, Leicester Square.**—The income was the lowest for the past thirteen years, but the year closed with a credit balance of £62; this surplus takes no account of a capital expenditure of £243 towards mortgage redemption. Of the total ordinary income, £4,422, patients' payments accounted for £2,059. Since the beginning of the war nearly a thousand sailors and soldiers suffering from skin diseases have been treated.

**St. Monica's Home Hospital for Sick Children, Brondesbury.**—The number of in-patients was 178, as against 156 in the previous year. The total income was £1,433, and the total expenditure £1,408. From the photograph in the report it will be gathered that a number of quite young babies are amongst the patients.

**St. Peter's Hospital, Covent Garden.**—The total income, £4,733, includes a legacy of £25 and £2,488 payments by patients. The total expenditure was £4,848. The average weekly cost of each in-patient has increased from £2 0s. 11d. to £2 3s. 1d.

### Meetings and Functions.

British Home and Hospital for Incurables, Streatham: A Garden Gathering at the Home, Saturday, July 1, 3.30 to 6 p.m.

South London Hospital for Women, South Side, Clapham Common.—Opening of the hospital by H.M. The Queen, Tuesday, July 4, 1916, 3 p.m. Visitors not admitted after 2.30 p.m.

Queen Charlotte's Lying-in Hospital, Marylebone Road.—Half-yearly general meeting at the hospital, Tuesday, July 4, at 3 p.m., Viscount Portman (the President) in the chair.

### Matrons' and Sisters' Appointments.

City Hospital, West Heath, Birmingham.—Miss K. Sheridan has been appointed sister. She was trained at Cork North Charitable Infirmary, and has since been sister in charge of Cavan County Infirmary and sister in charge of the out-patient department at Birmingham and Midland Hospital for Women. Miss Sheridan has also done private and military nursing.

Isleworth Infirmary.—Miss B. M. Wiltshire has been appointed ward sister. Trained at Kingston-upon-Hull Infirmary, she was later ward sister and night superintendent there. Miss Wiltshire holds the certificate of the Central Midwives Board.

Ochil Hills Sanatorium, Milnathort.—Miss Isabel McGrouther has been appointed matron. She received her training at Leeds Union Infirmary, and has since been charge nurse at the Grove Fever Hospital, Tooting, S.W.; sister at St. Leonard's Infirmary, Shoreditch; home sister and assistant matron at Belvidere Hospital, Glasgow; and deputy-matron at the City Hospital South, Liverpool.

**NOTE.—Particulars of Appointments for insertion in this column will be welcomed.**

### Gifts to Hospitals.

Sir William James Thomas, of Cardiff, has intimated his intention of endowing a bed at Netley Hospital, to be called the "Bethania Bed."

Mr. E. C. A. Leigh, for many years lower master at Eton, who died on April 3, left £500 to King Edward's Hospital Fund.

The Royal *Hamadryad* Seamen's Hospital, Cardiff, has received £1,000 from Mr. W. H. Seager, the Cardiff shipowner, and his wife for the endowment of a memorial bed.

The late Mr. Frank Daniels has bequeathed £25,000 (free of death duties) to the Lord Mayor of London for the time being, to be distributed by him among such charitable institutions and objects as he may in his absolute discretion select.

### THEATRICAL GARDEN PARTY.

The committee in charge of the arrangements for the Theatrical Garden Party at the Botanic Gardens on July 11 sends us the following:

"It was Madame Pavlova who wrote in the visitors' book of an hotel, 'I dance because I must,' and it was Joe Coyne, following hard upon her heels, who hurriedly scrawled, 'I sing because I can't.' Madame Pavlova will not be present at the Theatrical Garden Party, which is to be held at the Royal Botanic Gardens on July 11, but Joe Coyne will be 'on deck' prepared to meet all comers.

"Joe (we beg his pardon, Joseph) has a part-interest in 'Bohemia,' and those who know him will readily wager that the 'Kiosk' will have to live up to its name or show the reason why. Supporting Mr. Coyne will be found such 'stars' as Mr. Robert Hale, Mr. W. H. Berry, Miss Cicely Debenham, Miss Constance Hyem, Miss Peggy Rush, and a host of others. We have an uneasy suspicion that American drinks will be obtainable in 'Bohemia'—they usually are. As a mixologist Mr. Coyne bows only to Charley Mahoney, of the Hoffman House, and if asked for a 'Maiden's Blush' we make no doubt that he will produce the goods.

"It will cost precisely 3s. to test Mr. Coyne's abilities or disabilities as a 'bar-keep,' and it looks to us like an experiment 'weel worth the trying.' Tickets may be obtained at any theatre or library for 3s. in advance, or 5s. upon the day."

RATES OF SUBSCRIPTION: Twelve months, 6/6; Six months, 4/-; Three months, 2/-, post free to any address in the United Kingdom. Abroad, Twelve months, 8/8; Six months, 5/-; Three months, 2/6, post free. THE HOSPITAL "Index" to Volume LIX., October 1915 to March 1916, is now ready, price 1½d. per copy, post free. Address The Manager, "THE HOSPITAL," The Hospital Building, 28/29 Southampton Street, Strand, London, W.C.

